# Supplementary material for: Impact of Forage Sources on Ruminal Bacteriome and Carcass Traits in Hanwoo Steers During the Late Fattening Stages
Source: Microorganisms. 2024 Oct 17;12(10):2082. doi: 10.3390/microorganisms12102082 (PMC11510489; doi:10.3390/microorganisms12102082)
Supplement: Supplementary file 1 [file microorganisms-12-02082-s001.zip › microorganisms-3243308-supplementary.pdf]

Table S1. Dominant unclassified bacterial taxa in the ruminal bacteriota of Hanwoo steers fed different forage sources

Bacterial taxon		Forage sources Relative abundance (%)							
**Family**	**Dominance**	**OAT**	**RYE**	**IRS**	**BAR**	**RSS**	**Pooled SEM**	**LDA score**	***p*-value**
Lachnospirales UCF	RSS	0.189	0.516	0.021	0.137	**0.585**	0.463	3.501	0.0377
F082	IRS	0.268	0.085	**1.206**	0.741	0.167	0.996	3.784	0.0377
UBA929	OAT	**0.101**	0.028	0.047	0.096	0.035	0.064	2.807	0.0452
**Genus**	**Dominance**	**OAT**	**RYE**	**IRS**	**BAR**	**RSS**	**Pooled SEM**	**LDA score**	***p*-value**
Atopobiaceae UCG	RSS	0.087	0.205	0.029	0.018	**0.488**	0.205	3.402	0.0418
Lachnospirales UCG	RSS	0.189	0.516	0.021	0.137	**0.585**	0.463	3.458	0.0377
DSXL01	IRS	0.269	0.060	**0.391**	0.206	0.147	0.170	3.274	0.0374
Dethiosulfovibrionaceae UCG	RSS	0	0.139	0	0.051	**0.172**	0.162	3.029	0.0479
WRAI01	OAT	**0.101**	0.028	0.047	0.096	0.035	0.064	2.705	0.0452
Bifidobacteriaceae UCG	IRS	0	0	**0.090**	0	0	0.041	2.845	0.0080
XBD2001	OAT	**0.053**	0	0.015	0	0	0.018	2.732	0.0165
CAG-603	RYE	0	**0.042**	0.002	0	0.003	0.037	2.601	0.0337
**Species**	**Dominance**	**OAT**	**RYE**	**IRS**	**BAR**	**RSS**	**Pooled SEM**	**LDA score**	***p*-value**
UBA1067 sp004551905	BAR	0.111	0.070	0.229	**0.688**	0.009	0.183	3.555	0.0392
Atopobiaceae UCS	RSS	0.087	0.205	0.029	0.018	**0.488**	0.205	3.352	0.0418
UBA1067 sp900320855	BAR	0.132	0.095	0.357	**0.425**	0.021	0.276	3.346	0.0459
Lachnospirales UCS	RSS	0.189	0.516	0.021	0.137	**0.585**	0.463	3.418	0.0377
Limimorpha sp900318085	IRS	0.268	0.085	**1.206**	0.741	0.167	0.996	3.664	0.0377
Anaerobutyricum UCS	OAT	**0.253**	0.009	0.076	0.121	0.067	0.137	3.065	0.0414
Shuttleworthia UCS	IRS	0.075	0	**0.285**	0.049	0	0.394	3.110	0.0423
UBA2450 sp902800025	BAR	0	0.009	0	**0.062**	0	0.055	2.641	0.0274
DSXL01 sp011367845	IRS	0.269	0.060	**0.391**	0.206	0.147	0.170	3.242	0.0374
Butyrivibrio A 168226 sp000421405	IRS	0	0.012	**0.151**	0	0	0.082	2.870	0.0184
Dethiosulfovibrionaceae UCS	RSS	0	0.139	0	0.051	**0.172**	0.162	2.905	0.0479

Butyrivibrio A 168226 sp900116875	OAT	**0.061**	0	0.012	0	0	0.047	2.580	0.0212
WRAI01 sp009780275	OAT	**0.101**	0.028	0.047	0.096	0.035	0.064	2.571	0.0452
Bifidobacteriaceae UCS	IRS	0	0	**0.090**	0	0	0.041	2.730	0.0080
XBD2001 sp900116395	OAT	**0.053**	0	0.015	0	0	0.018	2.530	0.0165
Anaeroplasma UCS	RSS	0	0.008	0	0	**0.020**	0.014	2.287	0.0387
Ga6A1 UCS	RSS	0	0	0	0	**0.021**	0.014	2.402	0.0080
UBA2813 sp900319365	RYE	0.005	**0.112**	0.06	0.033	0.002	0.158	2.774	0.0391

UCF, unclassified family; UCG, unclassified genus; UCS, unclassified species
Dominance of each taxon for specific forage feeing were additionally shown as bold and underlined numbers.
OAT, oat hay; RYE, rye silage; IRS, Italian ryegrass silage; BAR, barley forage; RSS, rice straw silage
